# Association between organic nitrogen substrates and the optical purity of d-lactic acid during the fermentation by *Sporolactobacillus terrae* SBT-1

**DOI:** 10.1038/s41598-024-61247-4

**Published:** 2024-05-08

**Authors:** Sitanan Thitiprasert, Phetcharat Jaiaue, Nichakorn Amornbunchai, Jesnipit Thammakes, Jirabhorn Piluk, Piroonporn Srimongkol, Somboon Tanasupawat, Nuttha Thongchul

**Affiliations:** 1 Center of Excellence in Bioconversion and Bioseparation for Platform Chemical Production, Institute of Biotechnology and Genetic Engineering, Chulalongkorn University, Phayathai Road, Wangmai, Pathumwan, Bangkok, 10330 Thailand; 2 Institute of Biotechnology and Genetic Engineering, Chulalongkorn University, Phayathai Road, Wangmai, Pathumwan, Bangkok, 10330 Thailand; 3 Program in Biotechnology, Faculty of Science, Chulalongkorn University, Phayathai Road, Wangmai, Pathumwan, Bangkok, 10330 Thailand; 4 Department of Biochemistry and Microbiology, Faculty of Pharmaceutical Sciences, Chulalongkorn University, Phayathai Road, Wangmai, Pathumwan, Bangkok, 10330 Thailand

**Keywords:** *Sporolactobacillus terrae*, d-lactic acid, Optical purity, d-lactate dehydrogenase, Lactate racemase, Biochemistry, Biotechnology, Systems biology

## Abstract

The development of biotechnological lactic acid production has attracted attention to the potential production of an optically pure isomer of lactic acid, although the relationship between fermentation and the biosynthesis of highly optically pure d-lactic acid remains poorly understood. *Sporolactobacillus terrae* SBT-1 is an excellent d-lactic acid producer that depends on cultivation conditions. Herein, three enzymes responsible for synthesizing optically pure d-lactic acid, including d-lactate dehydrogenase (D-LDH; encoded by *ldh*Ds), l-lactate dehydrogenase (L-LDH; encoded by *ldh*Ls), and lactate racemase (Lar; encoded by *lar*A), were quantified under different organic nitrogen sources and concentration to study the relationship between fermentation conditions and synthesis pathway of optically pure lactic acid. Different organic nitrogen sources and concentrations significantly affected the quantity and quality of d-lactic acid produced by strain SBT-1 as well as the synthetic optically pure lactic acid pathway. Yeast extract is a preferred organic nitrogen source for achieving high catalytic efficiency of d-lactate dehydrogenase and increasing the transcription level of *ldh*A2, indicating that this enzyme plays a major role in d-lactic acid formation in *S. terrae* SBT-1. Furthermore, lactate racemization activity could be regulated by the presence of d-lactic acid. The results of this study suggest that specific nutrient requirements are necessary to achieve a stable and highly productive fermentation process for the d-lactic acid of an individual strain.

## Introduction

Lactic acid is a chiral compound with two optical isomers: L(+)- and d-lactic acid. Lactic acid is a versatile chemical that is widely used in food, pharmaceutical, cosmetic, and chemical industries^1^. Optically pure form of lactic acid (l- or d-isomer) is more valuable than racemic dl-lactic acid due to the industrial requirement of specific applications. The recent application of optically pure lactic acid is a monomer for synthesizing biodegradable and biocompatible plastics, polylactic acid (PLA). In general, a high optical purity of lactic acid (≥ 99%) is required for PLA production, and consequently, interest has grown in the microbial production of optically pure lactic acid^2^. Up to now, the fermentative process of optically pure l-lactic acid is extensively reported due to its widely used in the food industry, but economically fermentative production of d-lactic acid is still a challenging task.

The current limitations and challenges for efficient microbial production of optically pure d-lactic acid are focusing on the development of cost-effective fermentation process with a high yield and high optical purity of lactic acid^3^. In the fermentation process, substrate for lactic acid production has been significantly considered as a key factor to enable the cost-effective fermentative production of optically pure lactic acid. According to the literatures, yeast extract is mostly used for lactic acid fermentation with contributing the essential nutrients for both microbial growth and lactic acid production, although a price of yeast extract is one of the major factors in large-scale production^4^. Therefore, alternative the sources of nitrogen, for example corn steep liquor (CSL) have been extensively studied for cost-effective fermentation of lactic acid^5^. However, there is a little importance to address a stable and highly optical pure d-lactic acid fermentation process using inexpensive substrate approaches.

The genus *Sporolactobacillus* has been extensively reported as potential d-lactic acid producer that can produce high titers and optical purities of d-lactic acid^6–9^. According to the strains, several fermentation factors, including medium composition and growth conditions, can manipulate the enantiomeric ratio of lactic acid^10^, indicating that optically pure d-lactic acid production can be obtained by a specific strain with the proper fermentation conditions. In terms of the fermentative pathway, the optically pure l- and d-isomers of lactic acid are synthesized by NAD-dependent l-lactate dehydrogenase (L-LDH; EC 1.1.1.27) and NAD-dependent D-LDH (EC 1.1.1.28), respectively. Most lactic acid bacteria strains contain both L- and D-LDH, and these thereby influence the enantiomeric ratio of lactic acid. Additionally, the interconversion of lactate isomers can be occurred by lactate racemase activity (EC 5.1.2.1) which influences a racemic mixture of l- and d-lactic acid. Lactate racemase activity is recently reported in a few *Lactobacillus* species and is regulated by l-/d-lactic acid ratio^11,12^. However, the studies on the association of fermentation factors with mechanism of optically pure lactic acid synthesis pathway of *Sporolactobacillus* strains are scarce.

In our previous study, we reported the efficiency of the new isolate *S. terrae* SBT-1 for producing high d-lactic acid (up to 290 g/L) under high sugar concentration (up to 440 g/L). Three potential D-LDH-, two L-LDH-encoding genes (*ldh*Ds and *ldh*Ls), as well as a lactate racemase-encoding gene (*lar*A) which involved in optically pure lactic acid isomers were annotated from the whole-genome sequence of SBT-1^13^. However, it was noticed that enantiomeric ratio of d-lactic acid was influenced when varying fermentation substrates. Accordingly, the present study attempts to further investigate the influence of different nitrogen sources and concentrations on enzymatic activities and transcription level for enantiomeric changes of d-lactic acid by *S. terrae* SBT-1. Studies gaining the insight into the effect of both yeast extract and CSL with various concentrations on the synthesis pathway of highly optically pure d-lactic acid. The understanding of these phenomena could provide direction for the development of biotechnological d-lactic acid production.

## Materials and methods

### Bacterial strain and cultivation

*S. terrae* SBT-1 from our laboratory collection was used to evaluate enzymatic reaction, gene transcription, and d-lactic acid production. This bacterial strain exhibits superior activity in the fermentation of glucose or sucrose at extremely high concentrations into d-lactic acid^13^. A stock culture of *S. terrae* SBT-1 was maintained in 10% (w/v) skimmed milk and stored at − 80 °C. The working culture of SBT-1 was prepared from cells activated on a fresh glucose–yeast extract–peptone (GYP) agar slant containing 10 g/L glucose, 5 g/L yeast extract, 5 g/L peptone, 0.25 g/L KH_2_PO_4_, 0.25 g/L K_2_HPO_4_, 20 g/L agar, 5 g/L CaCO_3_, and 10 mL of salt solution, which contained 40 g/L MgSO_4_⋅7H_2_O, 2 g/L MnSO_4_⋅5H_2_O, 2 g/L FeSO_4_⋅7H_2_O, and 2 g/L NaCl. SBT-1 cells were cultured at 37 °C for 24 h anaerobically using a W-zip pouch containing AnaerobicPack-Anaero (Mitsubishi Gas Chemical). The culture was transferred onto the freshly prepared GYP agar slant every week. To begin fermentation, fresh culture slants were resuspended using 0.85% NaCl to reach an initial optical density at 600 nm (OD_600_) of approximately 0.3–0.4 for the preculture medium at 1% inoculum size.

### Influence of different organic nitrogen sources on d-lactic acid fermentation in *S. terrae* SBT-1

Three different organic nitrogen sources, including yeast extract, corn steep liquor (CSL), and urea, were tested their effect on d-lactic acid biosynthesis of *S. terrae* SBT-1 in shake-scale cultivation. All the tested nitrogen sources were used in quantities equivalent to the nitrogen content in 15 g/L yeast extract (1.62% (w/w)). Thus, the concentration of both CSL and urea were equal to 42 g/L and 4 g/L, respectively. The nitrogen content in the yeast extract, CSL, and urea were determined by the Kjeldahl method^14^.

The GY preculture medium (50 mL) was inoculated with 1% (v/v) cell suspension and anaerobically incubated at 37 °C and 200 rpm for 6 h. In the preculture step, the medium comprised of 10 g/L glucose, various organic nitrogen sources at the concentrations specified above, 4 g/L NH_4_Cl, 0.25 g/L KH_2_PO_4_, 0.25 g/L K_2_HPO_4_, 10 mL/L of salt solution, and 5 g/L CaCO_3_. In the fermentation step, the preculture broth was transferred to fermentation medium containing 120 g/L raw sugar at 50% inoculum size. Sterile 80 g/L CaCO_3_ powder was added for pH control. The fermentation broth (50 mL) was anaerobically incubated at 37 °C and 150 rpm for 48 h, after which it was collected to measure the growth of bacteria (OD_600_); concentrations of sugar, lactic acid, and byproducts; and optical purity of d-lactic acid.

### Influence of different concentrations of yeast extract and CSL on d-lactic acid fermentation

In order to compare the influence of different concentrations of yeast extract and CSL on the d-lactic acid production, the different amount of nitrogen contents was used as a criterion for experimental variation. The variation of 2 nitrogen contents, including 1.22% and 0.81% (w/w), which corresponded to 11.25 and 7.5 g/L of yeast extract and 31.5 and 21 g/L of CSL were conducted.

The 1% (v/v) bacterial cell suspension was inoculated into preculture medium containing 10 g/L glucose, varied concentrations of yeast extract or CSL, 4 g/L NH_4_Cl, 0.25 g/L KH_2_PO_4_, 0.25 g/L K_2_HPO_4_, 10 mL of salt solution, and 5 g/L CaCO_3_. The preculture broth was anaerobically incubated at 37 °C and 200 rpm for 6 h and then transferred into the fermentation media containing 120 g/L raw sugar and 80 g/L CaCO_3_ at 50% (v/v) inoculum size. The fermentation broth (50 mL) was anaerobically incubated at 37 °C and 150 rpm for 48 h. The fermentation broth was collected to measure the growth of bacteria (OD_600_); concentrations of sugar, lactic acid, and byproducts; and optical purity of d-lactic acid.

In addition to fermentation performance, the transcription levels of genes encoding L-LDH, D-LDH, and lactate racemase of *S. terrae* SBT-1 cultivated under different preculture medium were analyzed.

### Sample analysis

The collected fermentation broth was acidified using 1 M HCl to remove CaCO_3_ and then centrifuged at 10,000×*g* for 5 min to separate cell pellets and cell-free supernatant. Cell pellets were resuspended in 1 mL of deionized (DI) water and mixed thoroughly before measuring their OD_600_ values. The concentrations of remaining sugar and metabolites in the cell-free supernatant as well as the optical purity were measured using high performance liquid chromatography. To analyze the organic compounds, the cell-free supernatant was initially filtered through a hydrophilic polytetrafluoroethylene membrane and diluted with double-DI (DDI) water. The diluted sample was automatically injected into an Aminex HPX-87H ion exclusion organic acid column (300 × 7.8 mm; Bio-Rad, USA) and incubated at 45 °C. Sulfuric acid (0.005 M) was used as a mobile phase at a flowrate of 0.6 mL/min. Sucrose, glucose, fructose, lactic acid, acetic acid, and ethanol were detected using a refractive index detector. Standard solutions containing each component at concentrations of 0.25–2 g/L were automatically injected as the reference to determine sample concentrations. The optical purity of d-lactic acid was measured by injecting diluted cell-free samples into a chiral column (Sumipack, Sumichiral OA5000), which was maintained at 40 °C. A 0.001 M CuSO_4_ solution was used as the eluant at a flowrate of 0.8 mL/min. The UV detector was used to detect the lactate isomers at 254 nm. Standard solutions of d- and l-lactate were injected as the reference to determine the optical purity of the product. Product yield, productivity, and optical purity of d-lactate were assessed as previously described^15,16^.

### Enzymatic activity assays of *S. terrae* SBT-1

The changes in the activities of L-LDH, D-LDH, and lactate racemase, which are related to the production and optical purity of lactic acid, were determined using different nitrogen sources and total nitrogen contents in the preculture medium for d-lactic acid production of *S. terrae* SBT-1. Crude extract was prepared for use in enzyme assays as previously described^17^. The protein concentration of the crude extract was measured via the Lowry method, using bovine serum albumin as the standard.

The activity of LDH was assessed based on NADH oxidation at 340 nm. A 630-μL reaction mixture was prepared using 0.1 M phosphate buffer (pH 7.2 for D-LDH or pH 6.5 for L-LDH), 20 μL of 0.01 M NADH, 50 μL of 0.10 M sodium pyruvate, and 45 μL of DI water; the reaction was initiated by adding 15 μL of the cell extract at 37 °C and was monitored for 5 min. The absorbance of the reaction product was measured at 340 nm every 1 s for 20 min, and the rate of the decreasing absorbance at 340 nm per min was calculated. The activity of LDH was subsequently determined using the calibration plot of absorbance at 340 nm versus NADH concentration in micromoles. One unit of LDH indicated the amount of enzyme that transformed 1 μmol of NADH into lactic acid within 1 min at 37 °C^17^.

A commercial assay kit for lactate racemase (Cat. No.11112821035, Boehringer Mannheim, Rbiopharm) was employed. The assay solution comprising 500 μL of glycylglycine assay buffer, 100 μL of NAD+, 10 μL of glutamate–pyruvate transaminase, 10 μL of lithium l-lactate, 50 μL of crude extract, and 450 μL of DI water was incubated at 37 °C for 5 min, and the absorbance was detected at 340 nm. The reaction mixture was then supplemented with 10 μL of lithium d-lactate. The amount of NADH produced in the reaction was calculated using the calibration plot of absorbance at 340 nm vs. NADH concentration in micromoles. The reaction of equal amounts of l- and d-lactic acid led to the formation of NADH. The lactate racemase unit refers to the quantity of enzyme necessary to convert 1 μmol of l- or d-lactic acid to the other isomer within 1 min^17^.

### cDNA synthesis

Cell pellets for RNA extraction were isolated from the culture medium via centrifugation at 15,000 rpm and 4 °C for 15 min, after which they were washed thrice with sterile DI water. RNA was extracted using RNeasy mini kit (Qiagen, Germany) as previously described^18^. The purity and quality of the extracted RNA were verified using Nanodrop Spectrophotometer (DS-11FX+, DeNovix^®^, USA). The extracted RNA was converted to cDNA using Precision nanoScriptTM2 Reverse Transcription kit (Primer design, UK) and Biolab thermocycler (Eppendorf AG, Hamburg, Germany).

### Quantitative PCR

Quantitative PCR (qPCR) was employed to determine the transcriptional levels of genes encoding LDHs and lactate racemase under different cultivation conditions of *S. terrae* SBT-1 for d-lactic acid production. Based on the genomic analysis of *S. terrae* SBT-1 (GenBank under the accession number VCMU00000000), primers were designed for genes encoding three D-LDHs, two L-LDHs, and one racemase of *S. terrae* SBT-1 (Table 1). The same amount of cDNA of each sample was used to analyze the dynamic changes in gene expression via qPCR. Reaction mixtures were prepared in 0.1-mL individual low-profile tubes using 2× qPCRBIO SyGreen Mix Lo-ROX kit. Each reaction tube contained approximately 100 ng of cDNA and 1× qPCRBIO SyGreen Mix, 400 nM forward and reverse primers, and sufficient PCR DDI H_2_O to yield a 20-μL reaction mixture. The primers used for qPCR are shown in Table 1. Amplification was performed using a MyGo Pro Real-time PCR Cycler (IT IS Life Science Ltd., UK) based on a step program of denaturation for 2 min at 95 °C; followed by 45 cycles of 10 s at 95 °C, 30 s at 60 °C, and 10 s at 72 °C; and finally 60–97 °C for 1 min. qPCR was performed in triplicate for each sample. Then, the cycle threshold (Ct) values obtained from the program were normalized to those of the housekeeping gene *gapA* and were used to calculate the relative expression. The calculation of the comparative cycle threshold method was performed as described previously^19^.

**Table 1:** Oligonucleotide amplification, fragment length, and product sizes for qPCR.

Genes	Primer name	Primer sequence (5ʹ–3ʹ)	T_m_	%GC	Length (bp)	Product size (bp)
*hicD1*	hicD1_FP	ATGCGATTACGCAGTATTTCCA	58.3	41	22	120
	hicD1_RP	CACAATCGAATTGATCGGAAAC	58.0	41	22	
*ldh3*	ldh3_FP	ATGATTGGCGAACATGGTGAC	59.3	48	21	129
	ldh3_RP	TCCACCTGGTCACGGTCAAT	59.8	55	20	
*ldhA1*	ldhA1_FP	GGACTCGGTGCTAAAATCATCG	59.6	50	22	105
	ldhA1_RP	GTCCGCTTCTTTGAGTACCTCTTC	58.6	50	24	
*ldhA_1*	ldhA_1_FP	GCTGCGTTGAAGCAGAACTGT	59.3	52	21	148
	ldhA_1_RP	GGAAGGTGCAGGCTGACAATA	58.8	52	21	
*ldhA_2*	ldhA_2_FP	ACGGTGATCGCAAATGATATTG	58.5	41	22	120
	ldhA_2_RP	ATCCATAAGCGGCACATGAAG	58.7	48	21	
*larA*	larA_FP	GAAGCCCGCATTCGTATTCTC	59.7	52	21	128
	larA_RP	TCGTCTGTCGAAATGTGCATTAC	58.8	43	23	
*gapA*	gapA_FP	GCTGAAAACATCGTTCCTGCT	58.1	48	21	117
	gapA_RP	GGAACCTGATTTAACCGGAACA	58.8	45	22	

### Statistical analysis

Significant differences among triplicate data were assessed using SPSS software (IBM^®^ SPSS^®^ statistics version 22, USA). Data were compared via one-way analysis of variance. *p*-values of < 0.05 were considered to indicate statistical significance for all tests.

## Results and discussion

### Influence of different nitrogen sources on d-lactic acid production in *S. terrae* SBT-1

**Figure 1:**
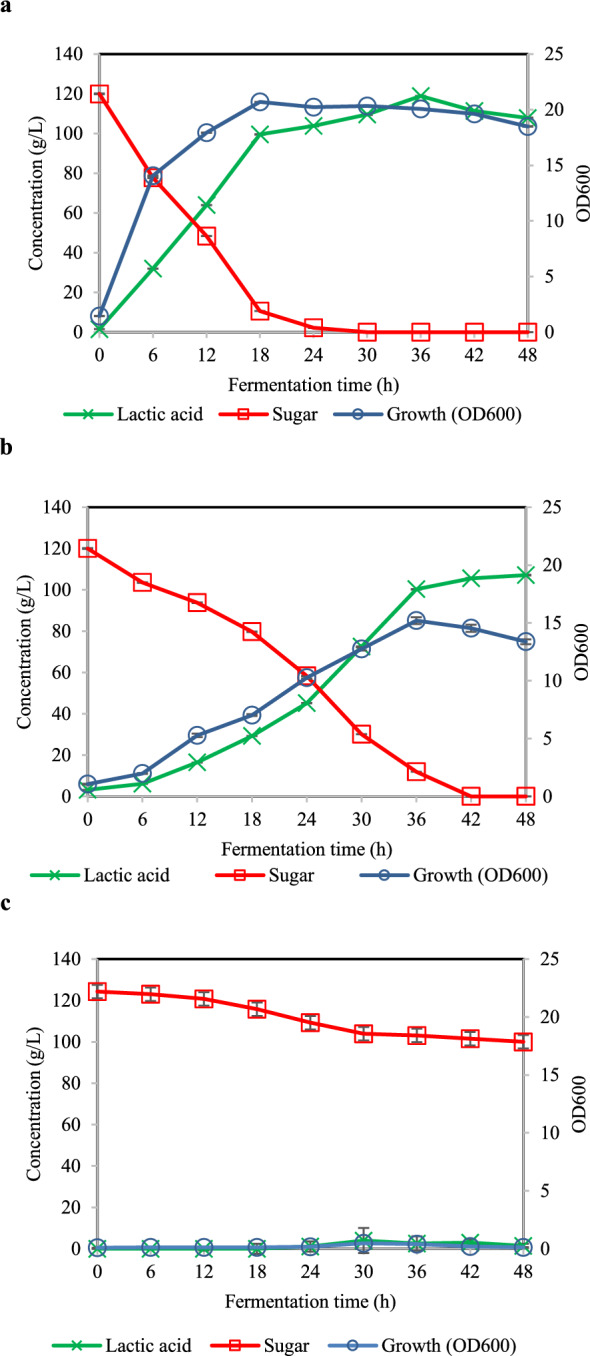
Fermentation profiles of *S. terrae* SBT-1 cultivated in preculture medium containing 15 g/L yeast extract (**a**), 42 g/L CSL (**b**), and 4 g/L urea (**c**). Experimental data are presented as mean values (n = 3).

Three different organic nitrogen sources, including yeast extract, CSL, and urea, were used to determine the effect of d-lactic acid fermentation performance in *S. terrae* SBT-1. All organic nitrogen sources were used at the same level of nitrogen content in 15 g/L yeast extract (1.62% (w/w) which corresponding to 42 g/L CSL and 4 g/L urea. Figure 1 presents the fermentation profiles of SBT-1 cultivated in preculture medium with different nitrogen sources. Urea negatively affected cell growth, resulting in the production of extremely low levels of lactic acid (Fig. 1c). Conversely, the use of complex nitrogen sources (either yeast extract or CSL) promoted cell growth and positively affected lactic acid fermentation in *S. terrae* SBT-1. Similar lactic acid concentration, yield, and productivity were observed when yeast extract or CSL were used as the nitrogen source at 120 g/L, 0.90 g/g, and 2.51 g/L h, respectively. In addition to the similar fermentation kinetic results, it was observed that yeast extract promoted bacterial growth more effectively than CSL, and sugar was completely consumed after 36 h of fermentation (Fig. 1a). Further, when CSL was used as a nitrogen source, sugar was completely consumed after 48 h of fermentation (Fig. 1b). Additionally, yeast extract showed a higher rate of sugar consumption (3.33 g/L h) and optical purity of d-lactic acid (99.99%ee), than CSL in preculture medium (Table 2). By this finding indicated that nitrogen content is not only essential component in d-lactic acid fermentation, the quality and quantity of nutrient sources contained in yeast extract and CSL play a crucial role on bacterial growth and lactic acid fermentation as well. Accordingly, both yeast extract and CSL were further used to examine the effect of their different concentrations on d-lactic acid production performance of *S. terrae* SBT-1.

**Table 2:** Fermentation kinetics of *Sporolactobacillus terrae* SBT-1 cultivated in preculture media containing different nitrogen sources.

Fermentation kinetics	Nitrogen source		
	Yeast extract (15 g/L)	CSL (42 g/L)	Urea (4 g/L)
Fermentation time (h)	36	48	–
Growth (OD_600_)	20.07 ± 0.15	13.37 ± 0.21	0.13 ± 0.02
Lactic acid concentration (g/L)	118.89 ± 0.11	107.15 ± 0.15	1.40 ± 0.56
Lactic acid yield (g/g)	0.99 ± 0.15	0.89 ± 0.00	0.05 ± 0.02
Lactic acid productivity (g/L h)	3.30 ± 0.00	2.23 ± 0.00	0.03 ± 0.01
Sugar consumption rate (g/L h)	3.33 ± 0.00	2.50 ± 0.00	0.47 ± 0.00
d-isomer (%ee)	99.99 ± 0.00	86.90 ± 2.72	–

All data are presented as the mean ± standard deviations of three replicates.

### Effect of different yeast extract and CSL concentrations on d-lactic acid production of *S. terrae* SBT-1

Both yeast extract and CSL were further used for determining their effect at different concentrations on d-lactic acid fermentation of *S. terrae* SBT-1. To determine an appropriate concentration of yeast extract and CSL, the variation of each nitrogen source was performed in the nitrogen content of 1.22% and 0.81% (w/w), corresponding to yeast extract concentration of 11.25 g/L and 7.5 g/L, respectively, and CSL concentration of 31.5 g/L and 21 g/L, respectively.

**Figure 2:**
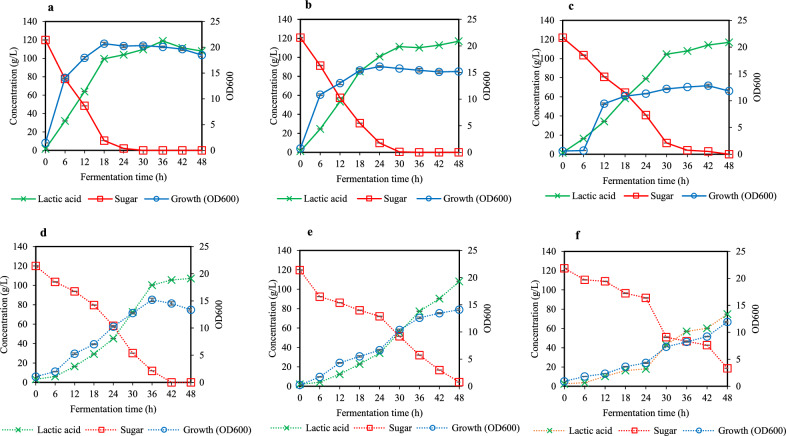
Fermentation profiles of *S. terrae* SBT-1 cultivated in preculture medium containing different nitrogen sources and concentrations. Preculture medium containing 15 g/L yeast extract (**a**), 11.25 g/L yeast extract (**b**), 7.5 g/L yeast extract (**c**), 42 g/L CSL (**d**), 31.5 g/L CSL, and 21 g/L CSL. Experimental data are presented as mean values (n = 3).

Figure 2 presents the fermentation profiles of *S. terrae* SBT-1 cultivated in preculture medium containing different nitrogen sources and concentrations. Decreasing the concentration of yeast extract from 15 to 7.5 g/L significantly reduced the fermentation performance of SBT-1 (Fig. 2a–c; Table 3). When 15 g/L yeast extract was used as the nitrogen source, sugar was completely consumed after 24 h of fermentation, with maximum fermentation kinetics of lactic acid production (Fig. 2a). While decreasing the concentration of yeast extract to 7.5 g/L, it was noticed that no significant change in lactic acid concentration but reducing bacterial growth was found, resulting in decreasing the rate of sugar consumption and lactic acid formation of *S. terrae* SBT-1 (Fig. 2b and c; Table 3). Furthermore, it was further found that the use of 15 g/L yeast extract could provide the production of d-lactic acid with approximately 100% enantiomeric purity, while decreased optical purity of d-lactic acid during fermentation was observed when the concentration of yeast extract was reduced (Table 3).

**Table 3:** Fermentation kinetics of d-lactic acid fermentation by *Sporolactobacillus terrae* SBT-1 cultivated in different preculture media.

Fermentation kinetics	Nitrogen source					
	Yeast extract (g/L)			CSL (g/L)		
	15	11.25	7.5	42	31.5	21
Fermentation time (h)	36	48	48	48	48	48
Growth (OD_600_)	20.07 ± 0.15	15.20 ± 0.10	11.81 ± 0.24	13.37 ± 0.21	14.10 ± 0.15	11.93 ± 0.08
Lactic acid concentration (g/L)	118.89 ± 0.11	117.01 ± 0.15	116.99 ± 0.02	107.15 ± 0.15	108.06 ± 0.21	74.84 ± 0.15
Lactic acid yield (g/g)	0.99 ± 0.15	0.98 ± 0.00	0.97 ± 0.00	0.89 ± 0.00	0.92 ± 0.00	0.69 ± 0.00
Lactic acid productivity (g/L h)	3.30 ± 0.00	2.44 ± 0.00	2.44 ± 0.00	2.23 ± 0.00	2.25 ± 0.00	1.56 ± 0.00
Sugar consumption rate (g/L h)	3.33 ± 0.00	2.50 ± 0.00	2.50 ± 0.00	2.50 ± 0.00	2.41 ± 0.00	2.11 ± 0.00
d-isomer (%ee)	99.99 ± 0.00	91.05 ± 0.02	88.19 ± 0.02	81.40 ± 0.23	82.68 ± 0.04	93.21 ± 0.05

All data are presented as the mean ± standard deviations of three replicates.

In the case of the media containing CSL, the decrease in the concentration of CSL from 42 g/L to 31.5 g/L did not result in a significant difference in the fermentation kinetics of d-lactic acid production in *S. terrae* SBT-1 (Fig. 2d and e; Table 3). As shown in Table 3, lactic acid concentration, yield, productivity, and sugar consumption rate obtained in the medium containing 42 g/L and 31.5 g/L CSL were 107.15–108.06 g/L, 0.89–0.92 g/g, 2.23–2.25 g/L·h, and 2.41–2.50 g/L·h, respectively. When the concentration of CSL was reduced at 21 g/L, bacterial cells could not incompletely consume substrate which caused the lowest lactic acid production in *S. terrae* SBT-1 (Fig. 2f and Table 3). In addition to the fermentation kinetics, the effect of CSL concentration on the enantiomeric purity of d-lactic acid was surprisingly observed. Reducing CSL concentration from 42 to 21 g/L, the optical purity of d-lactic acid was conversely increased. Thus, the optical purity of d-lactic acid was 81.40, 82.68, and 93.21%ee. in the medium based on 42, 31.5, and 21 g/L CSL, respectively (Table 3).

Based on this finding, yeast extract is still found as a preferable organic nitrogen source for d-lactic acid fermentation by the spore-forming lactic acid bacterium *S. terrae* SBT-1. This is due to the fact that yeast extract is a complex nutrient source that contains essential nutrients to promote bacterial growth, resulting in high levels of lactic acid production. In particular, yeast extract is rich in peptides, amino acids, vitamins, trace elements, and nucleic acids which enhance bacterial growth and lactic acid production^20,21^. However, yeast extract is recently considered as uneconomic nitrogen source for d-lactic acid production at industrial scale. Therefore, there have been various attempts to develop economic process of d-lactic acid production using low-cost materials, such as cottonseed meal^22^, peanut meal^8^, and CSL^23^. Nevertheless, most renewable materials cannot be directly utilized for lactic acid fermentation without pretreatment due to insufficient availability of free amino acids and small peptides, which results in a low fermentation performance of lactic acid^24^. As the previous study by Michalczyk et al.^25^, who examined various nitrogen sources, including inorganic and organic nitrogen sources, waste materials, food and agricultural products, for d-lactic acid production by *S. laevolacticus* DSM 442. Their results indicated that yeast extract was still the optimal choice for high levels of d-lactic acid production. In case of using alternative nitrogen source, increasing the nitrogen substrate content was suggested by prior enzymatic hydrolysis for achieving the effectiveness of lactic acid biosynthesis by bacterial strain^25^. Similar to Han et al.^6^, various agro-industrial wastes, such as peanut meal, soybean meal, corn steep liquor powder, and ammonium sulfate, were selected to substitute the usage of yeast extract as a sole nitrogen source for lactic acid production. They concluded that an efficient utilization of low-cost materials could be achieved by simultaneous enzymatic hydrolysis and fermentation approach^6^. Accordingly, the cost of pretreatment for lactic acid production utilizing alternative materials is one of the biggest challenges for fermentation process development, leading to a balance between nutrient costs and process efficiency. In addition to d-lactic acid production from alternative low-cost materials, investigation of individual nutritional requirements and to supplement the specific addition of essential nutrient sources (i.e., vitamins) have been intensively studied in lactic acid bacterial fermentation process^4,26,27^. Based on these pieces of information, it can be hypothesis that different nutrients present in yeast extract or CSL strongly influence on the metabolic lactic acid pathway of *S. terrae* SBT-1 (Additional file 1: Table 1). Furthermore, inappropriate amounts of nutrient sources contained in complex nitrogen source can negatively affect bacterial behavior, which is disadvantageous for the production of high-quality d-lactic acid. However, there are limited studies on the relationship of medium composition and metabolic response for d-lactic acid biosynthesis in this bacterial strain. For this reason, the relative catalytic efficiency of LDHs and lactate racemase under cultivation medium containing different yeast extract and CSL concentrations was further determined, and the transcription levels of *ldh*s and *lar*-encoded products were also analyzed.

### Influence of different yeast extract and CSL concentrations on the catalytic activity of key enzymes for the production of enantiomeric forms of lactic acid

#### LDH activity

**Figure 3:**
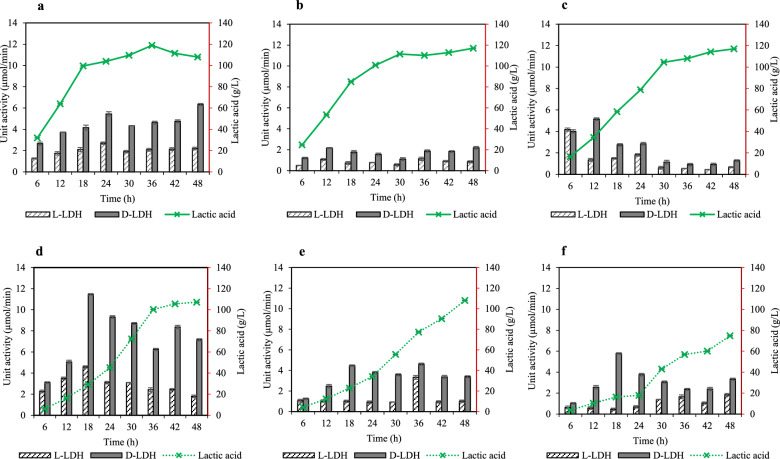
Lactic acid formation profiles and lactate dehydrogenase (L-LDH and D-LDH) activities during the fermentation phase of *S. terrae* SBT-1 under different nitrogen sources and concentrations. The tested nitrogen sources and concentrations were as follows: (**a**–**c**) yeast extract concentrations of 15, 11.25, and 7.5 g/L. (**d**–**f**) CSL concentrations of 42, 31.5, and 21 g/L. Experimental data are presented as mean values (n = 3).

The stereospecificity and optical purity of lactic acid are mainly depended on the conversion of pyruvate into l- and d-lactic acid by stereospecific NAD-dependent L-LDH and D-LDH, respectively. In this study, we determined the catalytic efficiencies of L- and D-LDH in SBT-1 cultivated in different media used for seed preparation. The result of L- and D-LDH activity during fermentation phase of SBT-1 cultivated in media containing different yeast extract and CSL concentrations indicated that D-LDH exhibits predominant activity under all conditions (Fig. 3). This finding indicated that *S. terrae* SBT-1 is a potential d-lactic acid producer.

The effect of yeast extract on the catalytic efficiency of both L- and D-LDH was examined at various yeast extract concentrations of 15, 11.25, and 7.5 g/L (Fig. 3a–c). Decreasing the yeast extract concentration affected the activity ratio of both L- and D-LDH. Thus, when 15 g/L yeast extract was used, D-LDH exhibited high enzymatic activity (Fig. 3a), whereas the use of a lower concentration of yeast extract induced L-LDH activity and consequently altered the catalytic efficiency of D-LDH (Fig. 3b,c). As shown in Table 3, a nonsignificant difference was observed in lactic acid concentration (116.99–118.89 g/L), whereas the optical purity of d-lactic acid significantly decreased from 99.99 to 88.19% when concentration of yeast extract was reduced from 15 to 7.5 g/L. This is because lactic acid configuration is regulated by stereospecific NAD-dependent enzymes such as L- or D-LDH, leading to the production of lactic acid with low optical purity (≤ 95%) or racemate level (dl-lactic acid)^28^. It is well known that yeast extract contains numerous essential amino acids (histidine and arginine), nonessential amino acids (aspartic acid and tyrosine), vitamins (B1, B2, and B6), and metal ions (Mg^2+^, Zn^2+^, and Mn^2+^) which are essential for the catalytic binding of both LDHs (Additional file 1: Table 1). Regarding the amino acids, histidine residues are highly conserved in the mechanism of bacterial LDHs^29,30^. These compounds have been widely reported as activators in the lactic acid biosynthesis pathway that promote bacterial cell growth and consequently produce high lactic acid titers^31,32^. In addition to amino acids, similar divalent metal ions (e.g., Ca^2+^, Mg^2+^, Mn^2+^, and Zn^2+^) are required for both enzymatic activities (D- and L-LDH). However, the presence of Fe^2+^ and K^+^ has a specific influence on catalytic L-LDH activity, whereas nickel ions (Ni^2+^) can induce D-LDH activity^33^. Therefore, it is possible that the negative effect on optical purity of d-lactic acid obtained from reducing the yeast extract concentration could occur because of the availability of nutrients in the yeast extract. Furthermore, diverse allosteric and catalytic functions of LDHs depend on the host species^30,34^. In previous reports on *S. inulinus* YBS1-5, D-LDH activity was induced by Ca^2+^ but slightly inhibited by Mg^2+^, whereas L-LDH activity was inhibited by Ca^2+^ but induced by Mg^2+^. Additionally, LDH activity was not affected by Na^+^ or K^+^^35^. Jia et al. reported that D-LDH activity of *Leuconostoc mesenteroides* was slightly enhanced by K^+^ but not by Mg^2+^, and fructose 1,6-biophosphate did not influence the enzymatic activity, indicating that D-LDH does not contain an allosteric site, similar to the structure of D-LDH from *Fusobacterium nucleatum* and *Pseudomonas aeruginosa*^29^.

**Figure 4:**
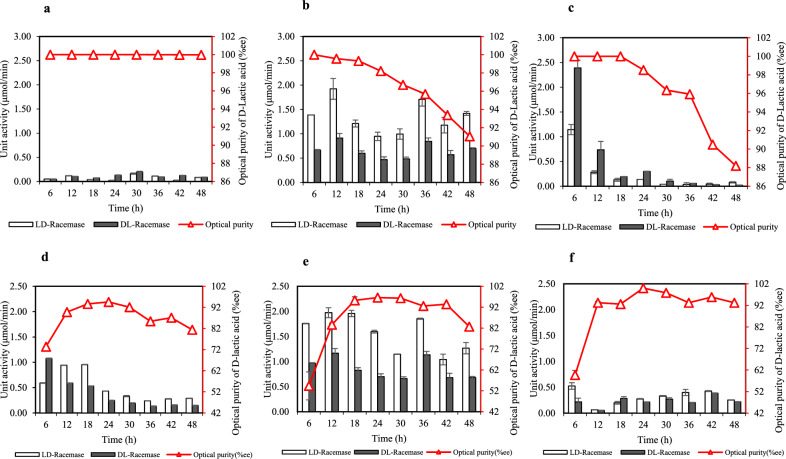
Optical purity of d-lactic acid and lactate racemase activities during fermentation phase of *S. terrae* SBT-1 under different nitrogen sources and concentrations. The tested nitrogen sources and concentrations were as follows: (**a**–**c**) yeast extract concentrations of 15, 11.25, and 7.5 g/L. (**d**–**f**) CSL concentrations of 42, 31.5, and 21 g/L. Experimental data are presented as mean values (n = 3).

In the case of CSL, decreasing the CSL concentration from 42 to 21 g/L significantly decreased lactic acid production but increased the optical purity of d-lactic acid (Table 3). The D-LDH activity was fully induced after 18–24 h of fermentation, during which the highest enantiomeric ratio of d-lactic acid could be obtained (Fig. 3d–f). Thus, a decrease in CSL concentration may regulate L-LDH activity and increase the enantiomeric ratio of d-lactic acid (Table 3; Fig. 4d–f). Compared with yeast extract, the essential nutrients in CSL are insufficient to promote the catalytic efficiency of D-LDH but can stimulate the catalytic activity of L-LDH (Additional file 1: Table 1). In particular, CSL lacks histidine and contains lower levels of vitamins, which play an important role in bacterial growth and lactic acid production, resulting in reduced fermentation performance of SBT-1. As mentioned above, both D- and L-LDH require similar types of metal ions. However, CSL does not contain Mn^2+^ or Zn^2+^, which may reduce enzymatic activity and subsequently decrease lactic acid production (Fig. 3). Bacterial allosteric L-LDH is commonly activated by fructose 1,6-bisphosphate. The conversion of fructose-6-phosphate into fructose 1,6-bisphosphate by phophofructokinase (PFK) requires Mg–ADP at the catalytic site to promote the binding affinity^17,36^. Therefore, the higher amounts of Mg^2+^ in CSL may be a factor that activates L-LDH through PFK.

#### Lactate racemase activity

The lactate racemization of l- and d-lactic acid by lactate racemase was also evaluated. The use of 15 g/L yeast extract resulted in considerable regulation of lactate racemase activity, producing d-lactic acid with stable optical purity of approximately 100% (Fig. 4a). Consequently, the decrease in yeast extract concentration (11.25 and 7.5 g/L) affected the interconversion of lactate isomers by lactate racemase, which is regulated by the ratio of l to d-lactic acid, resulting in reduced optical purity of lactic acid (Fig. 4b and c). In contrast, when the CSL concentration was reduced, the optical purity of d-lactic acid increased. According to previous research, lactate racemase activity is induced by the addition of exogenous l-lactic acid, but additional exogenous d-lactic acid cannot induce enzymatic activity^37^. However, based on the inducing effect of l-lactate that is counteracted by d-lactate, lactate racemase activity can be regulated at different ratios of l and d-lactic acid^38^. This finding is consistent with that reported in previous studies showing that the presence of l-lactic acid influences lactate racemase activity, which is involved in the interconversion of lactate isomers. As shown in Figs. 3d–f and 4d–f, when the CSL concentration was reduced, the synthesis of l-lactic acid decreased from 42 to 21 g/L, resulting in the regulation and counteracting of lactate racemase activity by d-lactic acid. Therefore, lactate racemase activity depends not only on the lactate isomer ratio but also on different nutritional compounds present in the cultivation medium, which may be critical influencers of lactate racemase activity. Lactate racemase requires Ni as a cofactor and is highly dependent on nicotinic acid to induce its catalytic activity^39^; thus, culture medium containing Ni and nicotinic acid (niacin or vitamin B3) can stimulate lactate racemization activity in SBT-1. Based on the nutritional profiles of yeast extract and CSL, only CSL contains niacin, indicating that lactate racemase activity could be regulated by removing exogenous nutritional compounds. Furthermore, the results confirmed that yeast extract is a more favorable medium than other nitrogen sources and could better manipulate the cellular metabolism of *S. terrae* SBT-1 for producing optically pure d-lactic acid.

### Dynamic changes in the transcription of genes encoding key enzymes responsible for producing optically pure lactic acid using different yeast extract and CSL concentration

To explore the influence of yeast extract and CSL concentrations on the optical purity of d-lactic acid produced by *S. terrae* SBT-1, qPCR was used to analyze the associated changes in the transcription of genes encoding key enzymes involved in d-lactic acid formation. The L- and D-LDH enzymes are encoded by *ldh*L and *ldh*D, respectively. However, although the enzyme lactate racemase responsible for the interconversion of lactate isomers has been reported in a few *Lactobacillus* strains, studies on the transcriptional level of this enzyme in *Sporolactobacillus* strains are scarce^11,35^. In our previous study, genome sequence annotation of *S. terrae* SBT-1 revealed two L-LDH-encoding genes (*hic*D1 and *ldh*3), three D-LDH-encoding genes (*ldh*A1, *ldh*A_1, and *ldh*A_2), and a lactate racemase-encoding gene (*lar*A)^13^.

**Figure 5:**
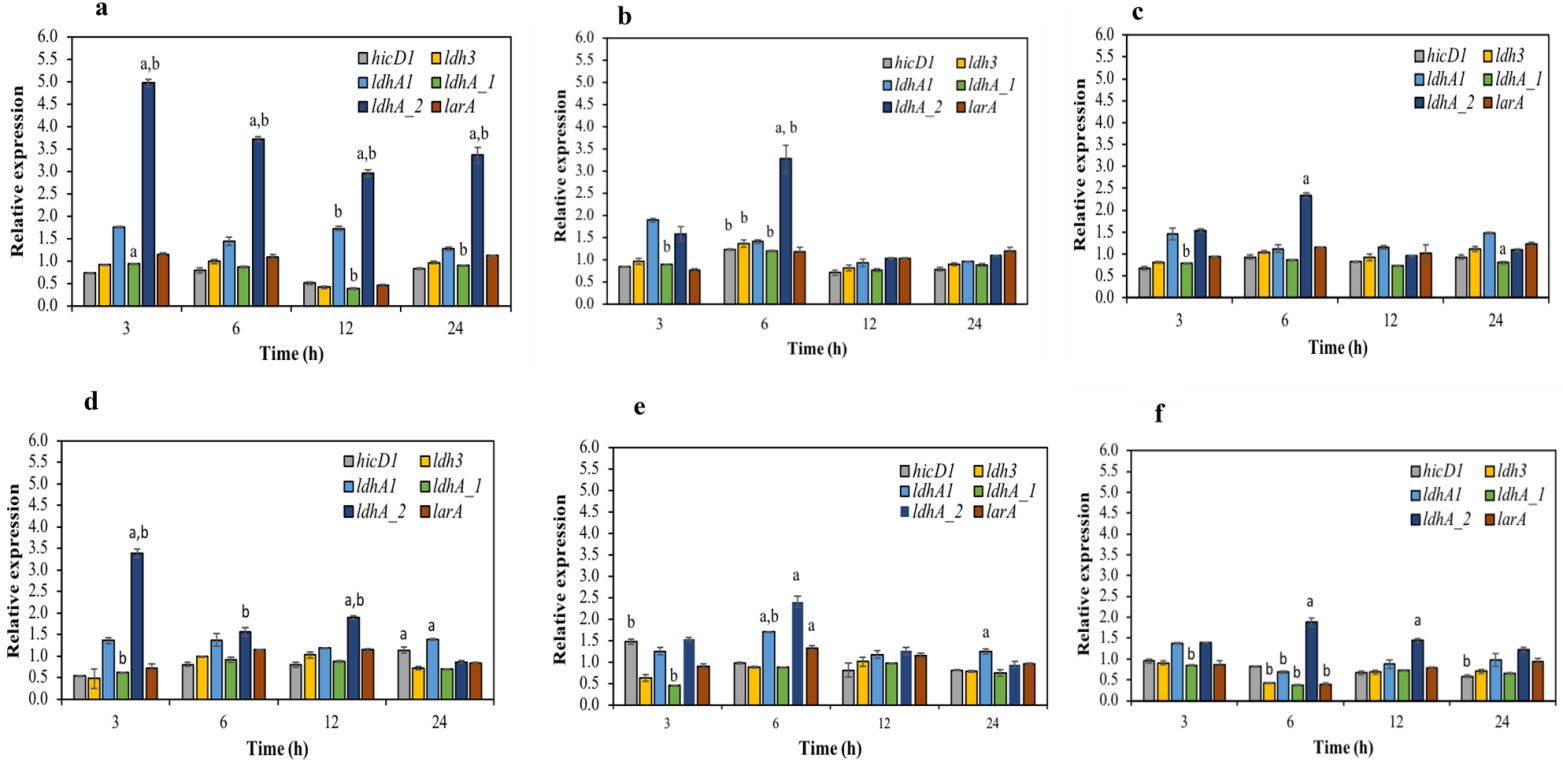
Gene expression dynamics of *hic*D1, *ldh*3, *ldh*A1, *ldh*A_1, *ldh*A_2, and *lar*A during cell cultivation. (**a**) 15 g/L yeast extract, (**b**) 11.25 g/L yeast extract, (**c**) 7.5 g/L yeast extract, (**d**) 42 g/L CSL, (**e**) 31.5 g/L CSL, and (**f**) 21 g/L CSL. Experimental data are presented as mean values (n = 3). ^a^Indicates a gene is significantly different from all genes, comparing with the same fermentation condition and sampling time. ^b^Indicates a gene is significantly different from all genes, comparing with different fermentation conditions.

Figure 5 shows the changes in the transcription levels of genes encoding L-LDH, D-LDH, and lactate racemase at different yeast extract and CSL concentrations in cultivation medium. The transcription level of *ldh*A_2 increased considerably in both yeast extract and CSL groups, and the use of 15 g/L yeast extract resulted in the highest transcription level of *ldh*A_2, consistent with the highest catalytic activity of D-LDH and enantiomeric ratio of d-lactic acid. These findings indicated that *ldh*A_2 plays a crucial role in the production of optically pure d-lactic acid in *S. terrae* SBT-1. However, the transcription level of each gene exhibited different patterns that depended on nitrogen source and concentration. Decreasing the yeast extract concentration influenced the transcription levels of L- and D-LDH-encoding genes, which were equivalent during the late exponential phase (12–24 h) (Fig. 5a–c). No significant changes were observed in the transcription levels of *lar*A at different yeast extract concentrations during the exponential fermentation phase (Fig. 5a–c). The transcription level of *lar*A was regulated by L-LDH-encoding genes (*hic*D1 and *ldh*3) and involved in *ldh*A_2 transcription level, indicating that the lactate racemase activity is affected by the l-/d-lactic acid ratio. In the use of cultivation medium-containing 42 g/L of CSL, the transcription level of ldhA_2 was highest at the initial stage of fermentation time (3 h) and then sharply decreased when the fermentation time reached at 24 h. Besides, the transcription levels of *hic*D1 and *ldh*3 were increased when the fermentation time entering in the logarithmic phase (6–12 h) (Fig. 5d). In contrast to the transcription level of genes encoding D- and L-LDH observed in 42 g/L CSL, decreasing the CSL concentration (31.5–21 g/L) showed the highest transcription level of ldhA_2 at 6 h of fermentation time with a greater the transcription level of genes encoding L-LDH (*hic*D1 and *ldh*3) throughout the exponential phase (6–24 h) (Fig. 5e,f). Accordingly, the optical purity of d-lactic acid produced in the reducing CSL concentration medium was higher than that of SBT-1 grown in CSL concentration of 42 g/L (Fig. 4d–f). Besides, it was further observed that the transcription level of *ldh*A_2 was decreased after 30 h of fermentation time in all different CSL concentrations (Fig. 5d–f), which correlated with the maximum optical purity of d-lactic acid was achieved at 24 h of fermentation time and consequently declined after 30 h of fermentation time (Fig. 4d–f). Thus, a corresponding optical purity of d-lactic acid was decreased from 94.58 to 81.40%, from 96.59 to 82.68%, and from 100 to 93.21% in the CSL concentration of 42 g/L, 31.5 g/L, and 21 g/L, respectively. Considering the transcription level of *lar*A, it was similarly determined using yeast extract, which revealed the relative transcription levels based on the dynamic changes in the transcription levels of L-LDH encoding genes (*hic*D1 and *ldh*3) (Fig. 5d–f).

These results were similar to those reported in previous studies on the transcriptional analysis of genes encoding LDHs in *S. inulinus*. They confirmed that the optical purity of d-lactic acid was regulated by the catalytic efficiency of D-LDH and transcriptional levels of *ldh*D-encoding genes, which correlated with different neutralizing agents^35^. Although the relative catalytic efficiency of the enzymes responsible for the optical purity of lactic acid produced by *Sporolactobacillus* strains is poorly understood, the regulation of lactate racemization activity at the transcription level has been reported in four *Lactobacillus* strains^11^. In a study by Singhvi et al., the transcription levels of D- and L-LDH in presence of diammonium hydrogen phosphate ((NH_4_)_2_HPO_4_) during d-lactic acid fermentation by *Lactobacillus* strains were reported. They found that the expression of LDH genes can be triggered by (NH_4_)_2_HPO_4_ with exhibiting higher d-lactic acid production along with l-lactic acid which depended on the strains^40^.

In addition to LDHs encoding genes, *lar*A-encoded lactate racemase plays a major role in catalyzing lactate racemization in lactic acid bacteria. Moreover, the lactate racemization system encoded by *lar*A is dependent on the l-/d-lactic acid ratio, which is induced by l-lactic acid and repressed by d-lactic acid^39,41^. The study results and recent knowledge regarding the enantiomeric selectivity of lactic acid isomers confirm that the l-/d-lactic acid ratio is a key physiological signal that regulates lactate racemase expression. This is based on the inducing effect of l-lactic acid that is counteracted by d-lactic acid. At a high d-/l-lactic acid ratio, d-lactic acid may suppress lactate racemization activity to produce d-lactic acid with high optical purity.

## Conclusions

Different organic nitrogen sources and concentrations directly affect the production of optically pure d-lactic acid by *S. terrae* SBT-1. d-lactic acid with optical purity of approximately 100%ee could be obtained using 15 g/L yeast extract as an organic nitrogen source in cultivation medium. To maximize the performance of bacterial strains for d-lactic acid production, the availability of sufficient nutrients is an essential factor for achieving optimal catalytic efficiency of both L-LDH and D-LDH. The high catalytic efficiency of D-LDH and elevated transcription level of *ldh*A_2 suggest that D-LDH plays a key role in d-lactic acid production by *S. terrae* SBT-1. Additionally, lactate racemization activity in SBT-1 could be regulated by d-lactic acid production. Thus, these results suggest that medium composition plays an important role in bacterial metabolic response and can support the establishment of an efficient fermentation platform for optically pure d-lactic acid.

### Supplementary Information

Supplementary Table S1.

## Data Availability

Data generated and analyzed in this study are included in the published article and the supplementary materials.

## Abbreviations

LDH: Lactate dehydrogenase
GYP: Glucose–yeast extract–peptone
CSL: Corn steep liquor
PLA: Polylactic acid
qPCR: Quantitative polymerase chain reaction
