# Supplementary material for: Association between organic nitrogen substrates and the optical purity of d-lactic acid during the fermentation by Sporolactobacillus terrae SBT-1
Source: Sci Rep. 2024 May 8;14:10522. doi: 10.1038/s41598-024-61247-4 (PMC11079031; doi:10.1038/s41598-024-61247-4)
Supplement: Supplementary file 1 — Supplementary Table S1. [file 41598_2024_61247_MOESM1_ESM.docx]

ADDITIONAL FILE

**Supplementary information for article**

**Association between organic nitrogen substrates and the optical purity of D-lactic acid during the fermentation by *Sporolactobacillus terrae* SBT-1**

**Submitted to Scientific Reports**

**Author and Corresponding author: Sitanan Thitiprasert**

**Institute of Biotechnology and Genetic Engineering, Chulalongkorn University, Thailand**

**E-mail:** [**sitanan.t@chula.ac.th**](mailto:sitanan.t@chula.ac.th)

**Table S1** ^a^Nutritional profiles of yeast extract and CSL

| Component | Yeast extract | CSL |
| --- | --- | --- |
| **Essential amino acid (g/100g)** |  |  |
| Histidine | 2.43 | N.D.^b^ |
| Hydroxylysine | N.D. | N.D. |
| Isoleucine | 3.22 | 0.47 |
| L-Arginine | 4.77 | 3.32 |
| Leucine | 5.45 | 1.93 |
| Lysine | 5.64 | 0.61 |
| Methionine | 1.24 | 1.03 |
| Phenylalanine | 2.84 | 0.76 |
| Threonine | 3.3 | 0.73 |
| Tryptophan | 0.67 | 0.03 |
| **Non-essential amino acid (g/100g)** | | |
| Valine | 3.81 | 0.98 |
| Aspartic | 6.68 | 1.14 |
| Cystine | 0.26 | N.D. |
| Glutamic acid | 9.50 | 2.80 |
| Glycine | 1.70 | 0.16 |
| Hydroxyproline | N.D. | 0.03 |
| L-Alanine | 4.85 | 2.73 |
| Proline | 3.03 | 3.22 |
| Serine | 3.30 | 0.99 |
| Tyrosine | 1.19 | 0.88 |
| **Vitamin (g/100 g)** |  |  |
| Biotin | 292 | 19.1 |
| Folic acid | 26.4 | N.D. |
| Glutamic acid (total) | 9.5 | N.D. |
| Pantothenic acid | 9.93 | 1.17 |
| Vit B1 | 2.67 | 0.66 |
| Vit B2 | 6.43 | 0.25 |
| Vit B3 (Niacin) | N.D. | 6.33 |
| Vit B6 | 0.96 | 0.73 |
| **Metal ion (mg/kg)** |  |  |
| Calcium | 384 | 257 |
| Copper | 1.2 | N.D. |
| Iron | 38.5 | 87.1 |
| Magnesium | 689 | 6400 |
| Manganese | 4.15 | N.D. |
| Nickel | 0.23 | 0.15 |
| Phosphorus | 25500 | 16110 |
| Potassium | 49008 | 25430 |
| Sodium | 939 | 70.3 |
| Sulfur | 5452 | 3640 |
| Zinc | 111 | N.D. |

^a^Nutritional profiles of yeast extract and CSL were determined by the service of ALS Laboratory Group (ALS Laboratory Group (Thailand), Co., Ltd.)

**^b^**N.D. = the component was not detected
